# Comparative efficacy of time-restricted eating combined with different exercise modalities for improving body composition, metabolic health, and physical performance in adults: a systematic review and fat-mass network meta-analysis

**DOI:** 10.3389/fnut.2026.1966901

**Published:** 2026-10-27

**Authors:** Hui Li, Cheng Lu, Gang Qin, Dacheng Li

**Affiliations:** 1 College of Arts and Information Engineering, Dalian Polytechnic University, Dalian, China; 2 Haihua College, Liaoning Normal University, Dalian, China; 3 Department of Sports Science, Hanyang University, Seoul, Republic of Korea

**Keywords:** aerobic exercise, concurrent training, high-intensity interval training, resistance training, time-restricted eating

## Abstract

**Background:**

Time-restricted eating (TRE) is commonly combined with exercise, but the relative effects of different exercise modalities remain uncertain.

**Objective:**

To compare usual diet or control, TRE alone, exercise alone, TRE plus aerobic exercise, TRE plus resistance training, TRE plus high-intensity interval training (HIIT), and TRE plus mixed or concurrent training.

**Methods:**

PubMed/MEDLINE, Web of Science, Scopus, Embase, the Cochrane Library, and Google Scholar were searched from 2016 to 20 July 2026. Seventeen reports representing 15 unique randomised trials met the eligibility criteria after cohort linkage. Eight independent trials contributed compatible fat-mass contrasts to a random-effects pairwise meta-analysis and a seven-node frequentist network meta-analysis.

**Results:**

TRE plus exercise reduced fat mass more than the same exercise without TRE [mean difference (MD) -1.85 kg, 95% confidence interval (CI) -2.34 to −1.35; *I*^2^ = 0%; *τ*^2^ = 0.00, and 95% prediction interval −2.46 to −1.23]. Network estimates versus usual diet or control were −3.32 kg for TRE plus concurrent training, −3.10 kg for TRE plus HIIT, −3.03 kg for TRE plus aerobic exercise, and −2.79 kg for TRE plus resistance training. Corresponding P-scores were 0.838, 0.757, 0.742, and 0.650. Confidence intervals for the active combined modalities overlapped. Residual network heterogeneity was not detected (Q = 3.29, df = 6, *p* = 0.772).

**Conclusion:**

TRE adds a modest reduction in fat mass when combined with exercise. Concurrent training, HIIT, and aerobic exercise ranked highly for fat-mass reduction, while resistance training had the most repeated evidence for preserving fat-free mass and strength.

**Systematic review registration:**

https://www.crd.york.ac.uk/PROSPERO/view/CRD420261440609, PROSPERO CRD420261440609.

## 1 Introduction

Intermittent fasting includes dietary approaches that extend the usual overnight fasting period. Time-restricted eating confines daily energy intake to a defined eating window and may influence energy intake, circadian alignment, and substrate use (1, 2).

Previous systematic reviews found modest reductions in body mass and fat mass when TRE was combined with exercise, with little evidence of a detrimental effect on fat-free mass (3–5). However, those reviews mainly pooled exercise modalities. They did not estimate the comparative effects of TRE combined with aerobic exercise, resistance training, high-intensity interval training (HIIT), or mixed and concurrent training.

This review compared seven prespecified intervention nodes: usual diet or control, TRE alone, exercise alone, TRE plus aerobic exercise, TRE plus resistance training, TRE plus HIIT, and TRE plus mixed or concurrent training. The primary quantitative outcome was fat mass in kilograms. Other primary outcomes were body weight, body mass index (BMI), body-fat percentage, waist circumference, and lean or fat-free mass. Secondary outcomes were fasting glucose, insulin, the homeostatic model assessment of insulin resistance (HOMA-IR), blood lipids, blood pressure, maximal oxygen uptake (VO₂max), muscular strength, and other physical-performance measures.

## 2 Materials and methods

### 2.1 Protocol, registration, and reporting

The protocol was prospectively registered in PROSPERO (CRD420261440609). Reporting followed PRISMA 2020 and the PRISMA extension for network meta-analysis (6, 7). Table 1 summarises the main reporting domains and their locations. The completed item-by-item PRISMA 2020 checklist is provided in Supplementary Table S1.

**Table 1:** PRISMA 2020 reporting summary.

Reporting domain	Summary in the main manuscript	Full documentation
Title, abstract and objectives	The title identifies the systematic review and NMA. The structured abstract reports the search date, independent trials, effect estimates, heterogeneity, rankings and conclusions.	Supplementary Table S1, items 1–4
Eligibility, search, and selection	Eligibility criteria, sources, dates, field concepts, deduplication, screening and grouped full-text exclusion reasons are reported in Methods and Results.	Supplementary Tables S1-S3
Data collection and trial linkage	Extraction fields and unique trial identifiers prevent double-counting of follow-up and secondary reports.	Supplementary Tables S4 and S11
Risk of bias and certainty	Outcome-level RoB 2 and GRADE/CINeMA methods and results are reported.	Supplementary Table S6; Table 7
Pairwise and network synthesis	Effect measures, random-effects pairwise methods, multi-arm handling, network estimates, rankings, sensitivity analyses and contribution are reported.	Supplementary Tables S5, S7-S10; calculation workbook
Registration and availability	PROSPERO registration and availability of the analytic workbook are stated.	Supplementary materials: Registration and data availability

The full item-by-item checklist is provided in Supplementary Table S1.

### 2.2 Eligibility criteria

Randomised parallel-group and randomised crossover trials were eligible when they enrolled adults aged 18 years or older; evaluated a daily TRE window of 12 h or less for at least 2 weeks; included a prospectively prescribed or standardised exercise programme; and reported at least one prespecified body-composition, metabolic-health, or physical-performance outcome. Healthy adults, adults who were overweight or obese, and adults with metabolic abnormalities were eligible. Ramadan fasting, alternate-day fasting, 5:2 fasting, acute fasted-versus-fed experiments, quasi-randomised studies, and conference abstracts without sufficient data were excluded.

### 2.3 Information sources and search strategy

The five core databases were PubMed/MEDLINE, Web of Science, Scopus, Embase, and the Cochrane Library. Google Scholar and backward and forward citation searching were Supplementary material. All sources were searched from 2016 to 20 July 2026. Table 2 gives the database-level summary, search role, and record count. The full line-by-line strategies, field tags, MeSH and Emtree terms, Boolean operators, and limits are provided in Supplementary Table S3.

**Table 2:** Summary of information sources and search strategies.

Source	Role	Search-strategy summary	Records
PubMed/MEDLINE	Core	TRE/feeding-window terms AND exercise/training terms AND randomised-trial terms; MeSH and title/abstract fields	372
Web of science	Core	Topic search for TRE terms, exercise modalities and randomi*/RCT/crossover terms	465
Scopus	Core	TITLE-ABS-KEY search for TRE terms, exercise modalities and randomised-trial terms; English articles	506
Embase	Core	Emtree and title/abstract/keyword terms for fasting/TRE, exercise and randomised trials; English articles	603
Cochrane library	Core	MeSH and title/abstract/keyword terms for TRE, exercise and randomised trials	207
Google scholar	Supplementary material	Focused title search for TRE, exercise and body-composition/metabolic/performance concepts	3,596

Complete reproducible strategies are provided in Supplementary Table S3.

Records were exported to a single reference library. Exact DOI, PMID, title, and author–year matches were removed first. Remaining records were deduplicated using normalised titles, first author, and publication year, followed by manual inspection. The two authors independently screened titles and abstracts, then assessed full texts against the prespecified criteria. Disagreements were resolved by consensus. One reviewer extracted data and the second reviewer checked all entries. Every excluded full-text report received one primary reason; grouped reasons are reported in Results, and the report-level list is provided in Supplementary Table S2.

### 2.4 Study selection, data extraction, and trial linkage

Extraction covered design, randomised and analysed sample sizes, population, eating window, exercise frequency, intensity, session duration, supervision, intervention duration, adherence, energy-intake control, measurement method, baseline and follow-up values, change scores, SDs, SEs, confidence intervals, and the exact source used for analysis.

Reports were linked under a unique trial identifier using registration number, study acronym, recruitment period, author group, sample size, and intervention arms. The Moro 2021 report was linked to the Moro 2016 cohort and was not treated as a separate randomised comparison (8, 9). The Haganes primary report and 2-year follow-up were linked under the TREHIIT trial identifier (10, 11). Only one prespecified time point from a cohort entered a given quantitative model.

### 2.5 Intervention nodes and outcome hierarchy

Usual diet or control comprised no TRE and no prescribed exercise. TRE alone comprised a daily eating window of 12 h or less without prescribed exercise. Exercise alone comprised the trial exercise protocol without TRE. Combined nodes were assigned according to the dominant prescribed exercise modality. Concurrent training required both aerobic and resistance components. High-intensity functional training was assigned to the mixed or concurrent node because it combined multiple functional exercise patterns. Exercise alone remained a single pooled node because too few modality-specific exercise-only trials were available to separate it further, unlike the TRE-combination nodes.

Fat mass, body-fat percentage and lean, or fat-free mass were prioritised as core outcomes. Fat mass in kilograms formed the connected network. Outcomes with compatible direct contrasts but no connected network were synthesised pairwise. Outcomes with incompatible scales or missing paired variance were synthesised in structured outcome tables without combining unlike measures.

### 2.6 Risk of bias

RoB 2 was applied at the outcome level using the official signaling questions and decision algorithms (12). Domains were the randomisation process, deviations from intended interventions, missing outcome data, measurement of the outcome, and selection of the reported result. Crossover trials were additionally assessed for period and carryover effects. Domain-level rationales and signaling-question records are supplied in Supplementary Table S6.

### 2.7 Statistical analysis

For pairwise synthesis, mean differences in change in fat mass were pooled with inverse-variance random effects. DerSimonian–Laird *τ*^2^, Cochran’s Q, *I*^2^, 95% CIs, and a t-based prediction interval were calculated (13). When a change-score SD was unavailable, it was derived from baseline and follow-up SDs with an assumed within-person correlation of 0.50. Sensitivity analyses used correlations of 0.30 and 0.70.

The frequentist network model used usual diet or control as the reference. Direct contrast equations were estimated by weighted generalised least squares. Multi-arm trials contributed correlated contrasts from the same randomisation. Shared-arm covariance was evaluated by sensitivity analyses, and no shared control group was entered as an independent duplicate. Relative effects, a league table, P-scores, rank probabilities and a contribution matrix were generated (14). Full model code and output are archived in the analytic workbook referenced in Table 1 for independent verification.

Transitivity was evaluated across age, sex, BMI status, training status, metabolic risk, eating-window duration, intervention duration, energy-intake control, exercise supervision, and body-composition method. The network contained multi-arm loops but no independent head-to-head loop between combined exercise modalities. Residual model fit was reported rather than treating within-trial multi-arm geometry as independent inconsistency evidence.

Analyses were performed in Python 3.11 using NumPy, SciPy, pandas, matplotlib, and NetworkX. All statistical tests were two-sided. A *p*-value below 0.05 was considered statistically significant.

### 2.8 Certainty of evidence

Certainty was assessed independently by the two reviewers, with disagreements resolved by consensus, using GRADE and CINeMA domains: within-study bias, reporting bias, indirectness, imprecision, heterogeneity, and incoherence (15, 16). Ratings were linked directly to the wording of the conclusions.

## 3 Results

### 3.1 Study selection

The searches identified 5,749 records. After removal of 1,880 duplicates, 3,869 records were screened, and 3,844 were excluded. Twenty-five database reports and six reports identified through citation searching and trial linkage were assessed in full. Fourteen reports were excluded: ineligible fasting regimen (*n* = 3), ineligible exercise/TRE intervention or intervention duration (*n* = 6), ineligible population or outcome (*n* = 3), and ineligible study design or publication type (*n* = 2). Seventeen reports representing 15 unique randomised trials were included. Eight trials contributed to the pairwise fat-mass analysis and the connected fat-mass network. Table 3 summarises the exclusion categories; the report-level reasons are provided in Supplementary Table S2 (Figure 1).

**Table 3:** Summary of full-text exclusion reasons.

Primary exclusion category	Reports, *n*	Summary
Ineligible fasting regimen	3	Alternate-day fasting, Ramadan fasting, or intermittent energy restriction rather than a daily TRE window
Ineligible exercise/TRE intervention or duration	6	No prescribed exercise comparator, acute exposure, exercise-timing study, or non-separable dietary co-intervention
Ineligible population or outcome	3	Pregnancy-specific population or no prespecified body-composition, metabolic-health or performance outcome
Ineligible study design or publication type	2	Quasi-randomised allocation or conference abstract without sufficient data

Individual report-level decisions are listed in Supplementary Table S2.

**Figure 1:**
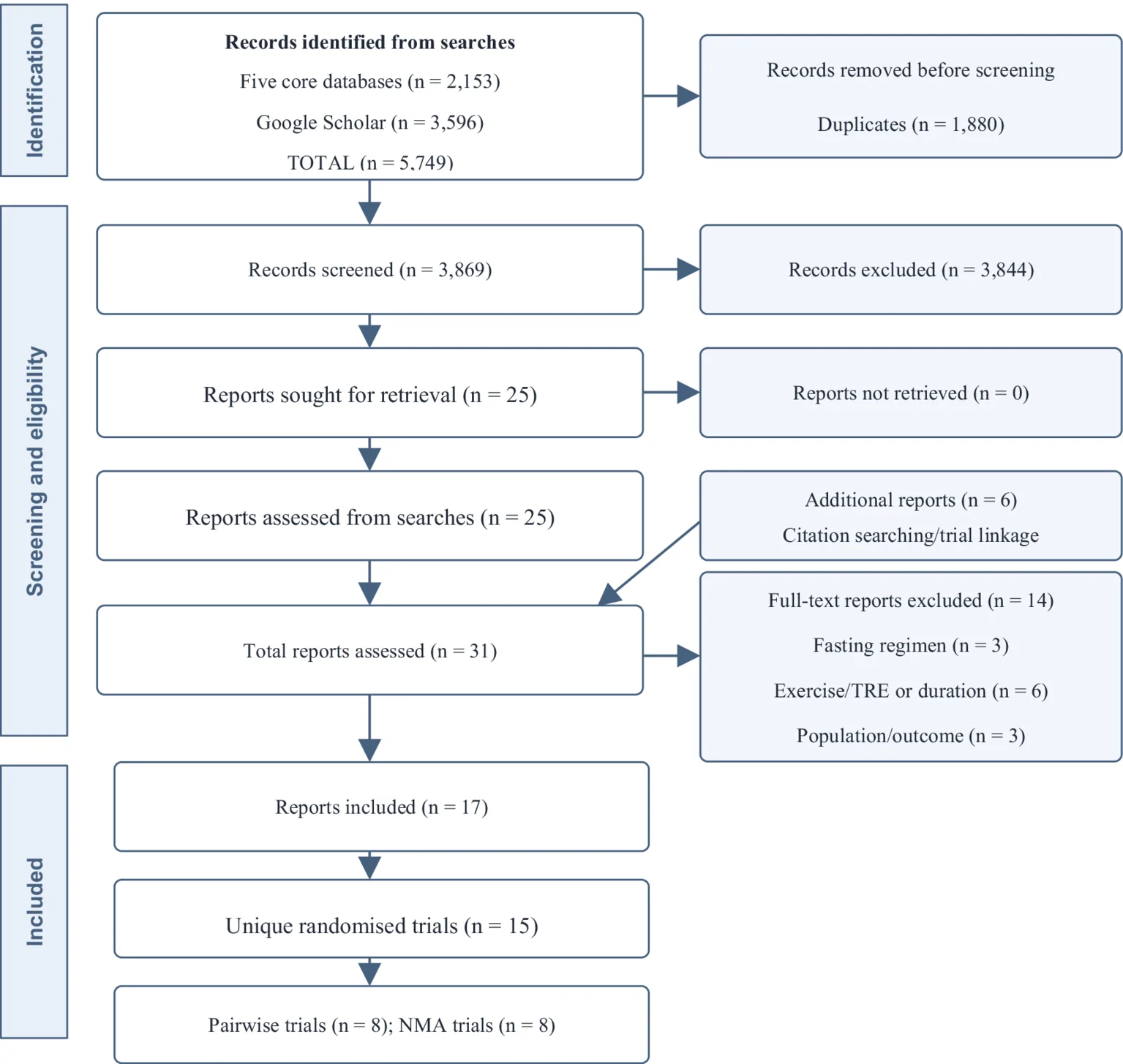
PRISMA 2020 flow diagram.

### 3.2 Included trials and report linkage

The included trials covered resistance training, endurance and aerobic exercise, walking, HIIT, and mixed or concurrent training. Intervention duration ranged from 4 to 12 weeks for primary trial endpoints, with linked follow-up to 12 months and 2 years. Trial linkage prevented double-counting of the Moro and TREHIIT cohorts (Table 4).

**Table 4:** Characteristics, report linkage and analytic use of the 15 unique trials.

ID	Trial/report linkage	Design and sample	Population	TRE and exercise	Duration	Use in synthesis
T1	Moro cohort; Moro 2016 primary; Moro 2021 long-term follow-up (8, 9)	Parallel RCT; 34 randomised; 20 at 12-month follow-up	Resistance-trained men	8 h daily; Resistance training, 3 sessions/week	8 weeks; linked 12-month follow-up	Primary 8-week report used in quantitative model; follow-up linked, not double-counted
T2	Tinsley 2017; Primary report (26)	Parallel RCT; 28 randomised; 18 analysed	Recreationally active men	4 h on 4 days/week; Resistance training, 3 sessions/week	8 weeks	Fat-mass change and imputed variance used
T3	Tinsley 2019; Primary report (17)	Three-arm RCT; 40 ITT; 24 per protocol	Resistance-trained women	=7.5 h daily; Supervised resistance training	8 weeks	ITT retained; no compatible arm-level variance for primary network
T4	Stratton 2020; Primary report (18)	Parallel RCT; 26	Recreationally active men	8 h daily; Supervised full-body resistance training	4 weeks	Fat-mass change used; change variance imputed
T5	Kotarsky 2021; Primary report (27)	Parallel RCT; 21 completers	Adults with overweight/obesity	12:00–20:00; Aerobic plus supervised resistance training	8 weeks	Fat-mass contrast used
T6	Moro 2020; Primary report (19)	Parallel RCT; 16	Elite male cyclists	10:00–18:00; Endurance cycling	4 weeks	Body-fat percentage and performance synthesis
T7	Brady 2021; Primary report (25)	Parallel RCT; 17	Male middle−/long-distance runners	12:00–20:00; Endurance running	8 weeks	Body composition and running outcomes
T8	Martínez-Rodríguez 2021; Primary report (28)	Randomised trial; Active women	Active women	TRE/IF protocol; HIIT	8 weeks	Body composition and performance synthesis
T9	TREHIIT; Haganes 2022 primary; Haganes 2025 follow-up (10, 11)	Four-arm RCT; 131 primary	Women with overweight/obesity	≤10 h daily; HIIT, 3 sessions/week	7 weeks; linked 2-year follow-up	Primary report used in network; follow-up linked
T10	Correia 2023; Primary report (20)	Randomised crossover; Adult men	Healthy trained men	8 h daily; Resistance training	4 weeks/condition	Paired synthesis; not entered without paired variance
T11	Correia 2024; Primary report (21)	Randomised crossover; 15 analysed	Male recreational runners	13:00–21:00; Structured endurance running	4 weeks/condition	Paired synthesis; not entered without paired variance
T12	Liu 2023; Primary report (29)	Four-arm RCT; 77	Female university students with hidden obesity	TRE protocol; Walking exercise	8 weeks	Body composition synthesis; exact compatible change variance unavailable
T13	Ameur 2024; Primary report (23)	Three-arm RCT; 64	Inactive women with obesity	≤8 h daily; HIFT, 3 sessions/week	12 weeks	Fat-mass contrast used with conservative imputed variance
T14	Cui 2025; Primary report (22)	Four-arm RCT; Young adults with overweight/obesity	Young adults with overweight/obesity	10 h daily; Resistance training	8 weeks	Reported fat-mass changes used
T15	Dai 2025; Primary report (24)	Four-arm assessor-blinded RCT; 104 ITT	Women aged 40–60 with overweight/obesity	Flexible 8 h daily; Aerobic exercise	12 weeks	Adjusted 99% CI contrasts used

### 3.3 Risk of bias

Four trials were judged at low risk of bias for the fat-mass outcome, and 11 had some concerns. The most frequent concerns related to incomplete reporting of allocation concealment, attrition or completer analysis, and the absence of a prespecified analysis plan for every reported body-composition outcome. Objective DXA, BIA, or multi-compartment measurements generally supported low risk in the outcome-measurement domain (Figure 2).

**Figure 2:**
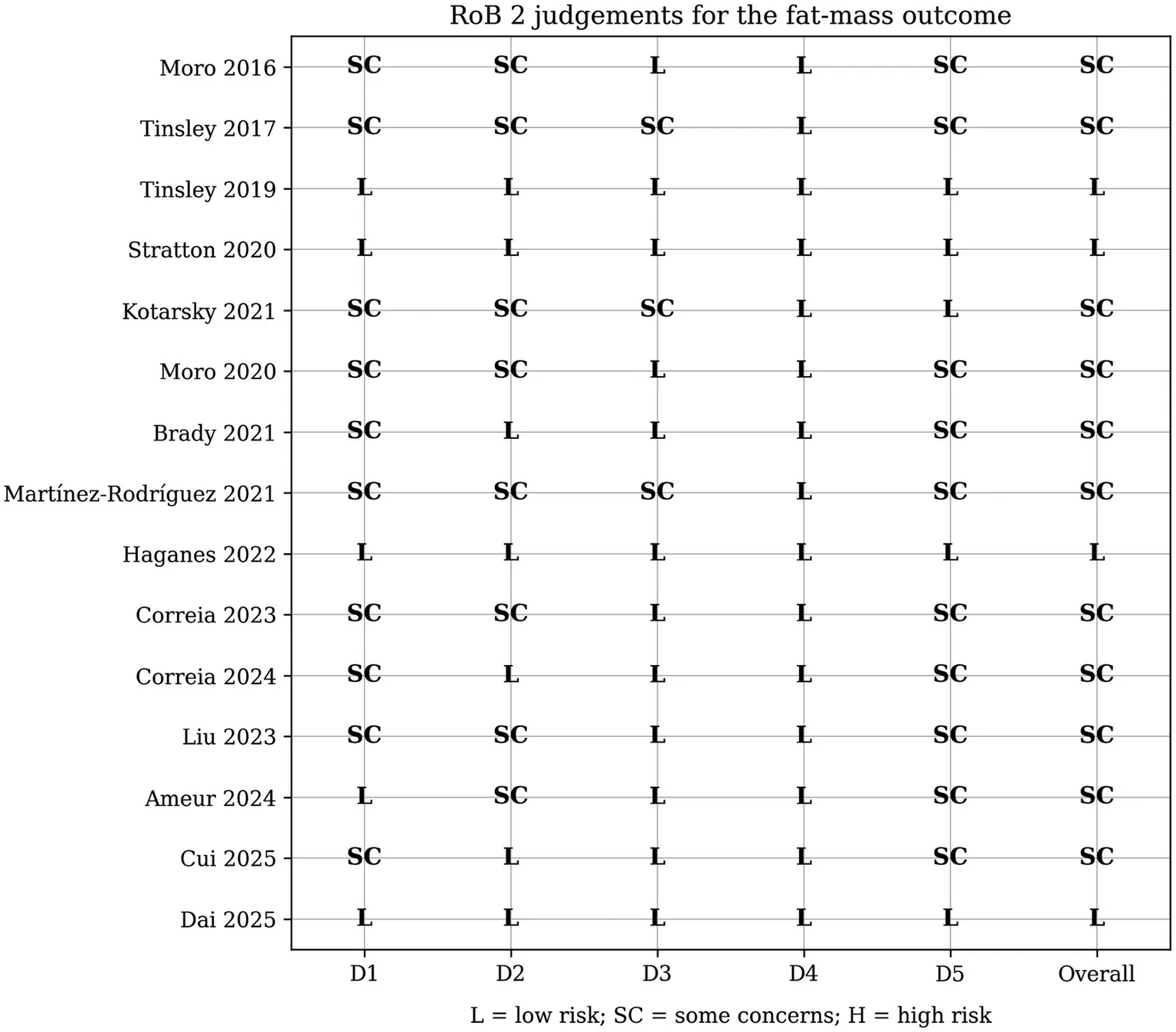
RoB 2 judgements for the fat-mass outcome. D1, randomisation; D2, deviations; D3, missing data; D4, outcome measurement; D5, selective reporting.

### 3.4 Pairwise meta-analysis of fat mass

Eight independent comparisons contributed to the pairwise model. TRE plus exercise reduced fat mass by 1.85 kg more than the same exercise without TRE (MD -1.85 kg, 95% CI -2.34 to −1.35). Heterogeneity was not detected (Q = 3.63, df = 7, *p* = 0.822; *I*^2^ = 0%; *τ*^2^ = 0.00). The 95% prediction interval was −2.46 to −1.23 kg.

The result was stable when the assumed baseline–follow-up correlation was changed from 0.30 to 0.70. Pooled estimates ranged from −1.86 to −1.84 kg (Figure 3).

**Figure 3:**
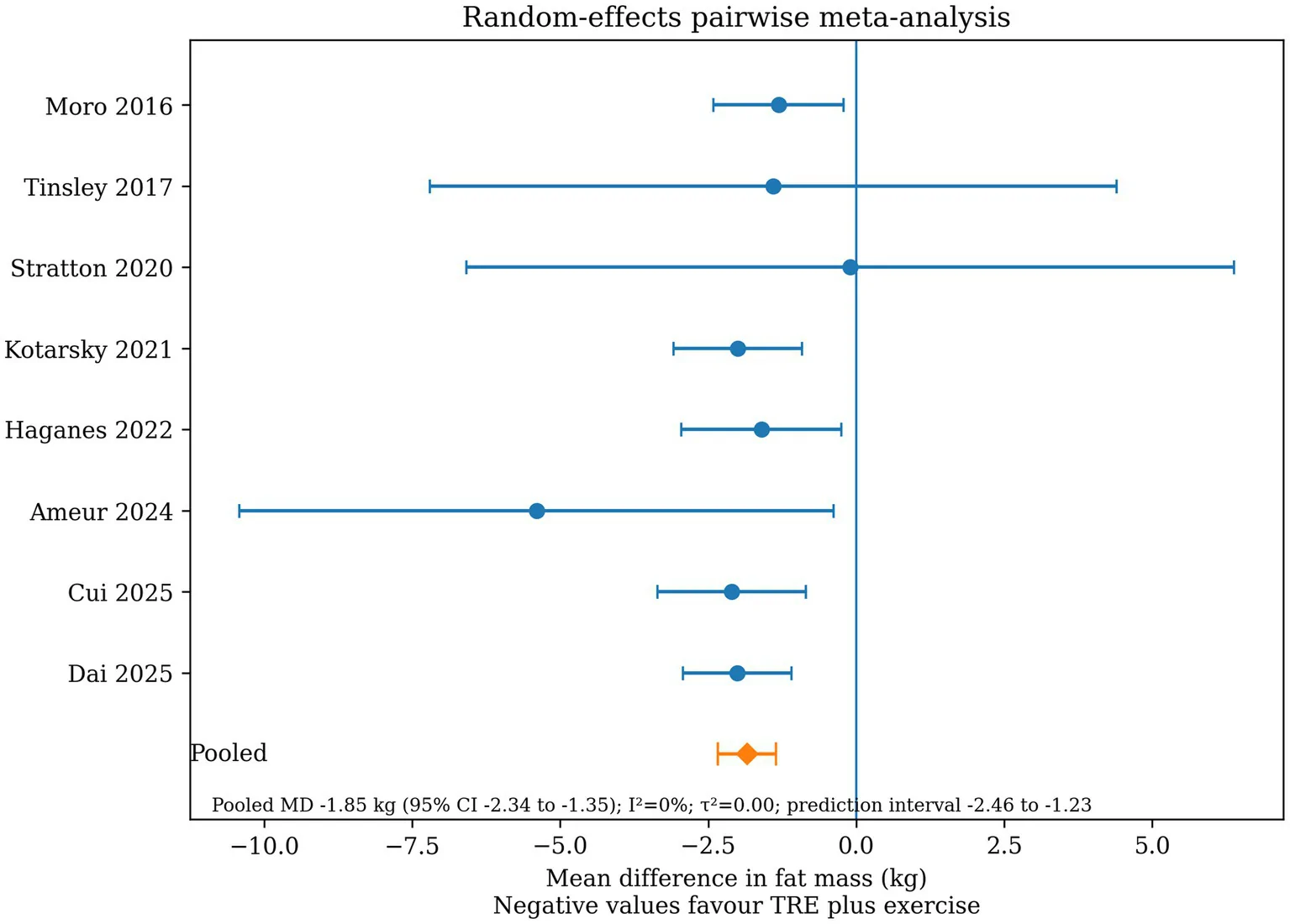
Random-effects pairwise meta-analysis of fat-mass change.

### 3.5 Evidence network and transitivity

The fat-mass network contained eight trials and seven nodes. Four resistance-training comparisons connected exercise alone with TRE plus resistance training. Two mixed or concurrent-training comparisons connected exercise alone with the concurrent node. The Dai and Haganes multi-arm trials connected control, TRE, exercise, and the aerobic or HIIT combined node (Figure 4).

**Figure 4:**
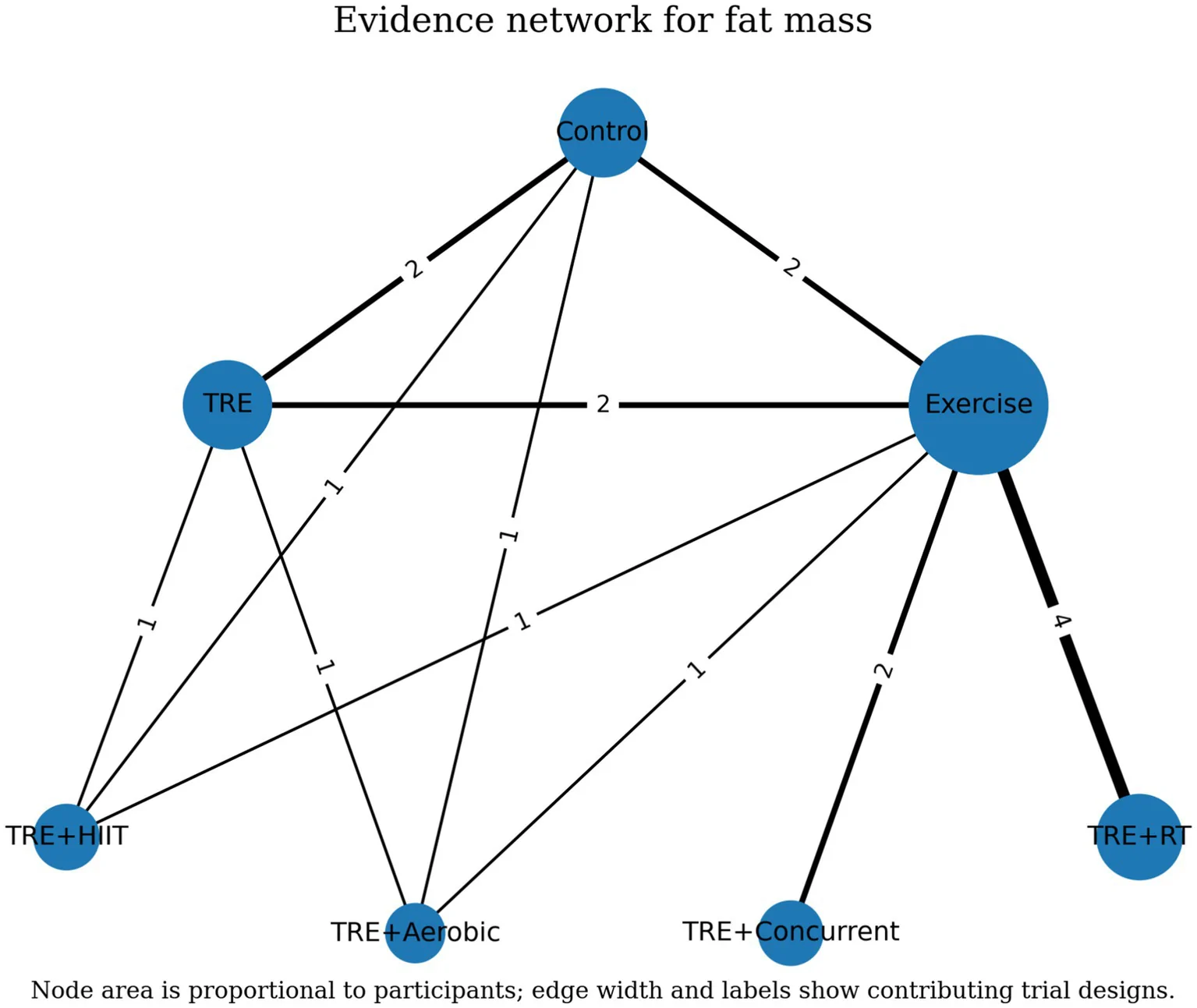
Evidence network for fat mass. Node area represents participants and edge width represents contributing trial designs.

Potential effect modifiers were unevenly distributed. Resistance-training trials primarily enrolled younger trained adults, whereas the aerobic, HIIT and mixed-modality trials more often enrolled women with overweight or obesity. This distribution was reflected in the certainty ratings and in the cautious interpretation of cross-modality rankings. As exercise alone pooled several modalities while TRE-combination nodes were modality-specific, indirect comparisons among the combined nodes should be interpreted cautiously; with only eight trials across seven nodes and no head-to-head trials between combined modalities, P-score rankings are indicative rather than definitive.

### 3.6 Network estimates, rankings, and contribution

All combined nodes had lower estimated fat mass than the usual diet or control. TRE plus concurrent training ranked first (P-score 0.838), followed by TRE plus HIIT (0.757), TRE plus aerobic exercise (0.742), and TRE plus resistance training (0.650). Confidence intervals for the combined modalities overlapped. Residual model heterogeneity was not detected (Q = 3.29, df = 6, *p* = 0.772; Figures 5–8; Table 5).

**Figure 5:**
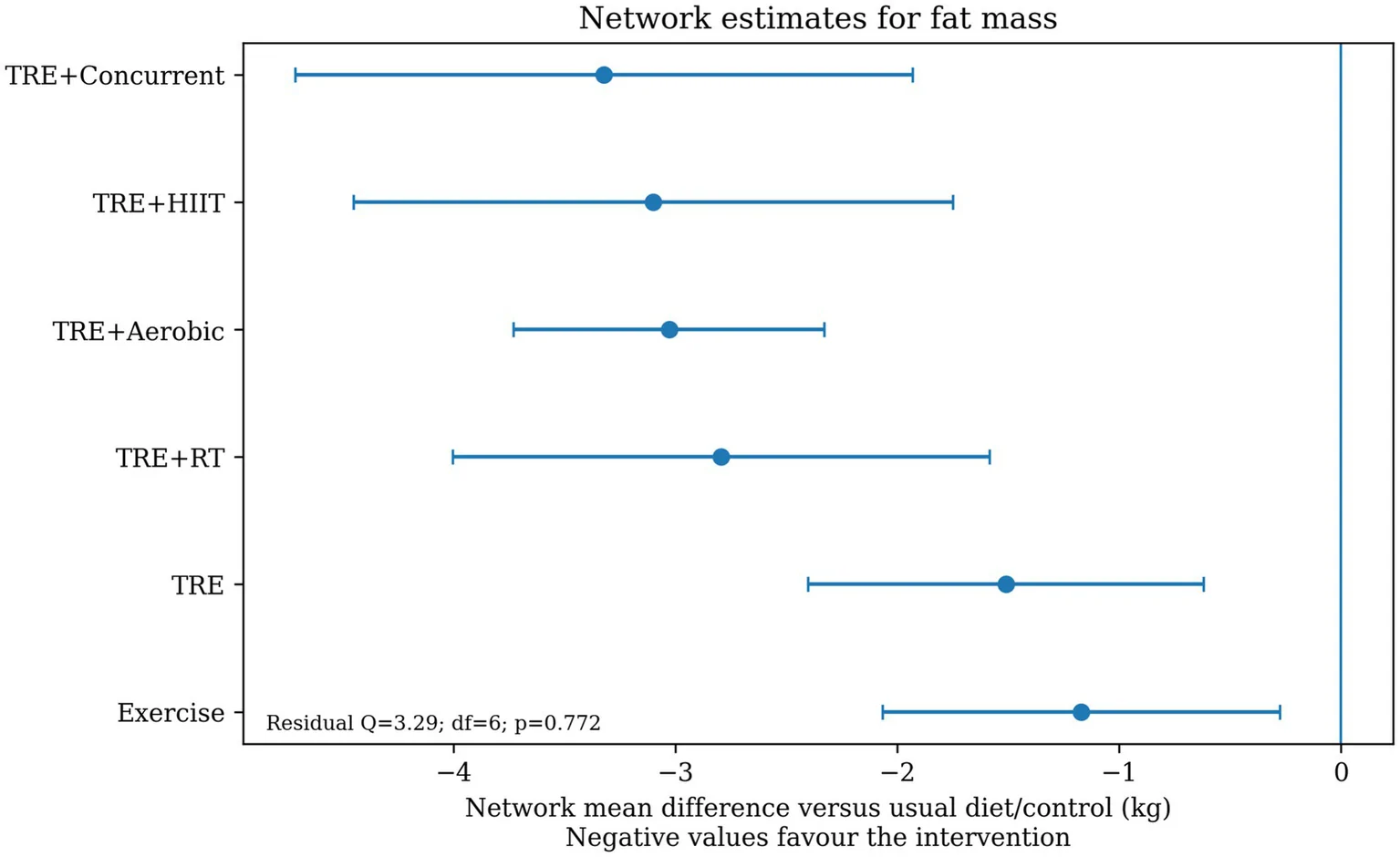
Network estimates for fat mass relative to usual diet or control.

**Figure 6:**
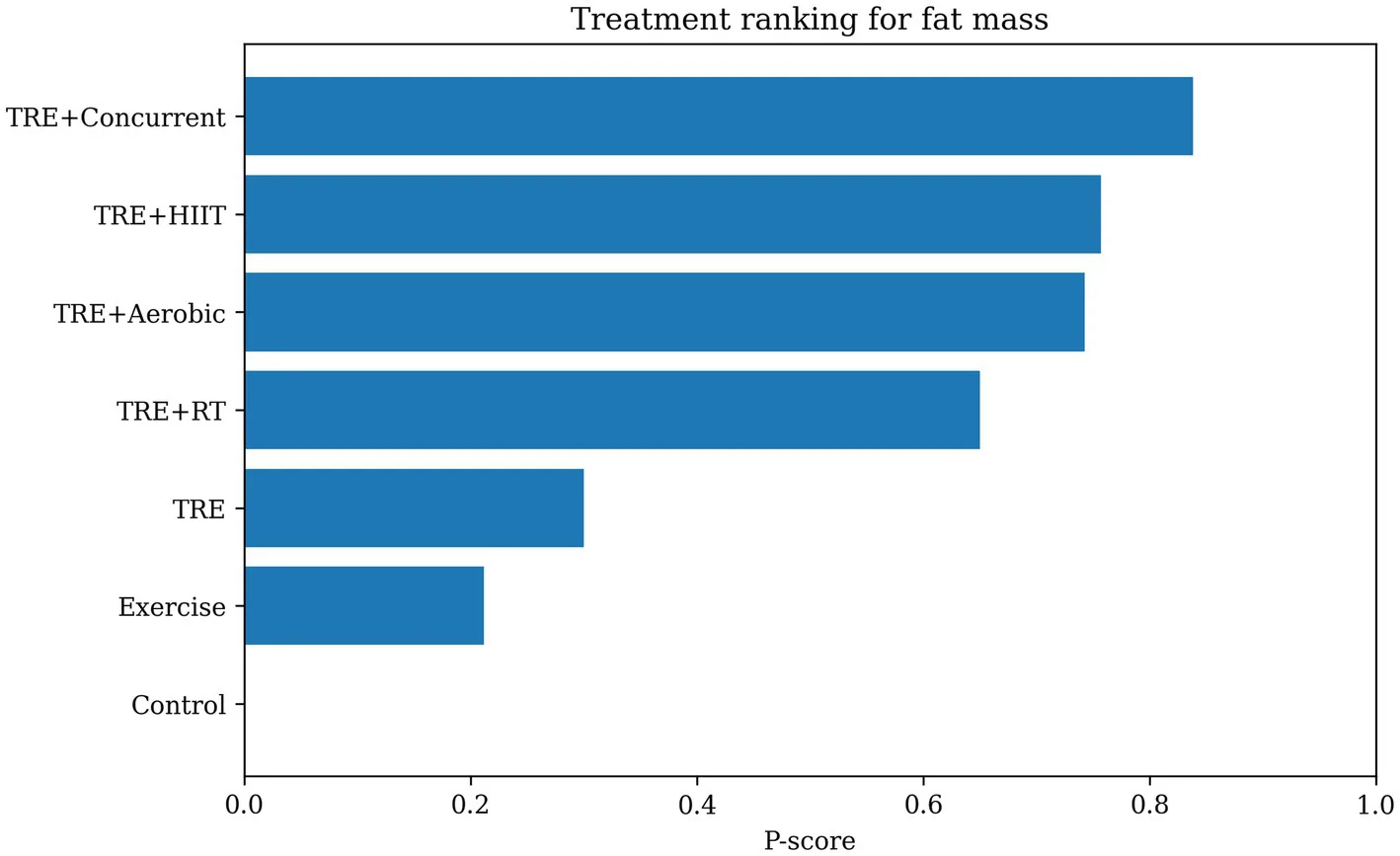
P-score ranking. Rankings describe probability and do not establish clinical superiority.

**Figure 7:**
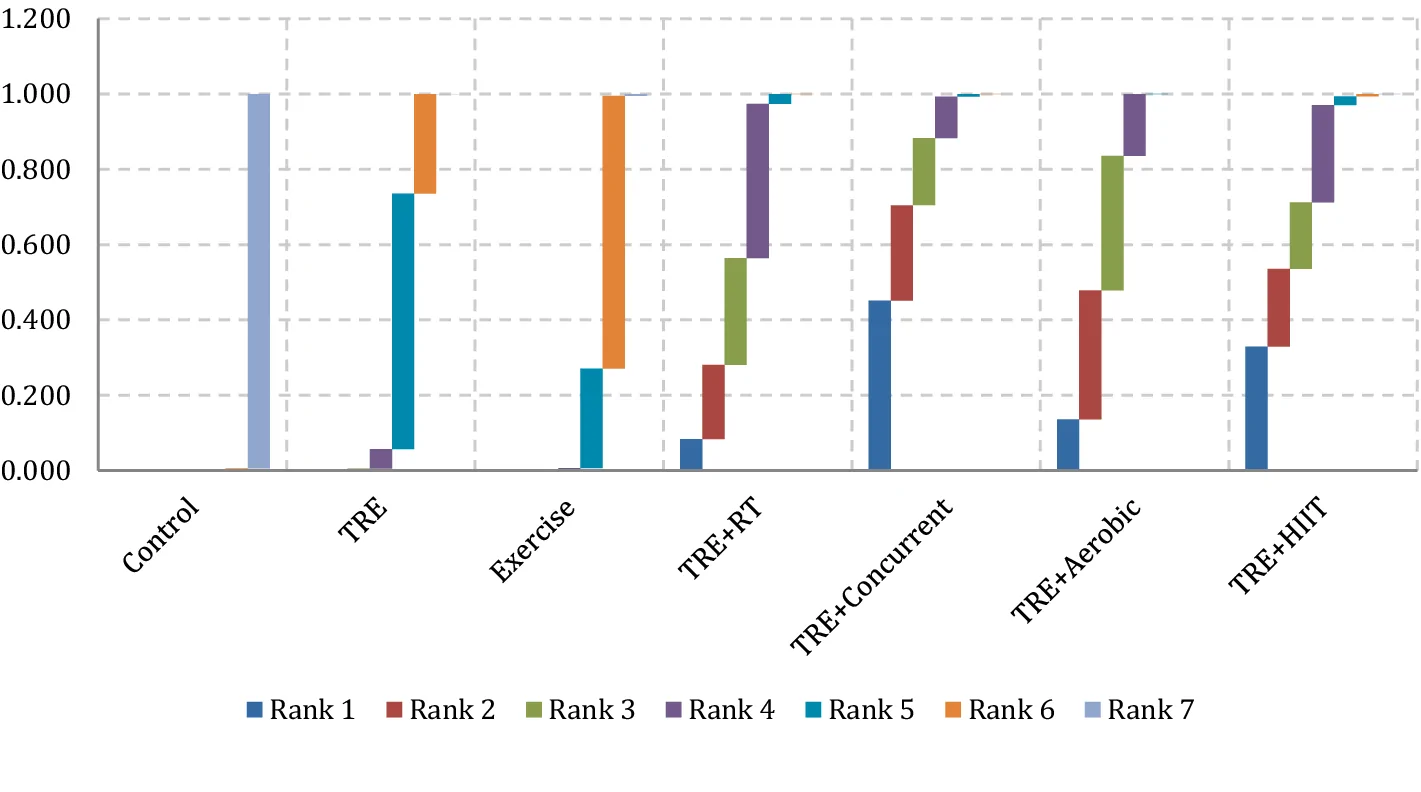
Rankogram for the seven intervention nodes.

**Figure 8:**
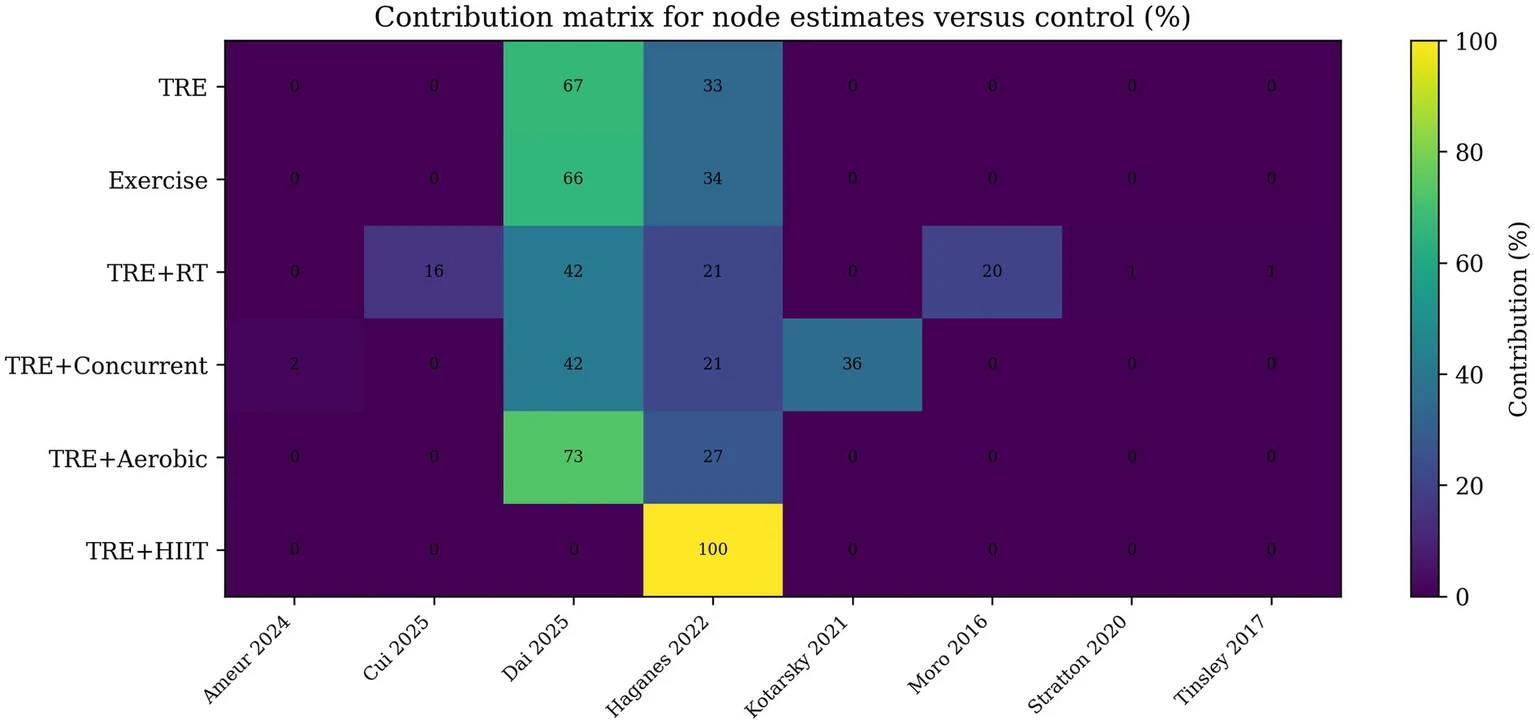
Contribution matrix for node estimates versus control. Values are percentage contributions derived from the weighted network design matrix.

**Table 5:** Network estimates and P-scores for fat mass.

Node	MD vs control, kg (95% CI)	*p*-score	Rank
TRE + Concurrent	−3.32 (−4.72 to −1.93)	0.838	1
TRE + HIIT	−3.10 (−4.45 to −1.75)	0.757	2
TRE + Aerobic	−3.03 (−3.73 to −2.33)	0.742	3
TRE + RT	−2.79 (−4.00 to −1.58)	0.650	4
TRE	−1.51 (−2.40 to −0.62)	0.300	5
Exercise	−1.17 (−2.07 to −0.27)	0.212	6
Control	Reference	0.001	7

Negative values favour the intervention.

### 3.7 League table

See Table 6 here.

**Table 6:** League table for fat mass.

Row treatment	Control	TRE	Exercise	TRE+RT	TRE+Concurrent	TRE+Aerobic	TRE+HIIT
Control		1.51 (0.62 to 2.40)	1.17 (0.27 to 2.07)	2.79 (1.58 to 4.00)	3.32 (1.93 to 4.72)	3.03 (2.33 to 3.73)	3.10 (1.75 to 4.45)
TRE	−1.51 (−2.40 to −0.62)		−0.34 (−1.40 to 0.72)	1.28 (−0.05 to 2.62)	1.81 (0.31 to 3.32)	1.52 (0.74 to 2.30)	1.59 (−0.03 to 3.21)
Exercise	−1.17 (−2.07 to −0.27)	0.34 (−0.72 to 1.40)	—	1.62 (0.81 to 2.44)	2.15 (1.09 to 3.22)	1.86 (1.07 to 2.65)	1.93 (0.31 to 3.55)
TRE + RT	−2.79 (−4.00 to −1.58)	−1.28 (−2.62 to 0.05)	−1.62 (−2.44 to −0.81)	—	0.53 (−0.81 to 1.87)	0.24 (−0.90 to 1.37)	0.31 (−1.51 to 2.12)
TRE + Concurrent	−3.32 (−4.72 to −1.93)	−1.81 (−3.32 to −0.31)	−2.15 (−3.22 to −1.09)	−0.53 (−1.87 to 0.81)	—	−0.29 (−1.62 to 1.03)	−0.22 (−2.16 to 1.72)
TRE + Aerobic	−3.03 (−3.73 to −2.33)	−1.52 (−2.30 to −0.74)	−1.86 (−2.65 to −1.07)	−0.24 (−1.37 to 0.90)	0.29 (−1.03 to 1.62)	—	0.07 (−1.45 to 1.59)
TRE + HIIT	−3.10 (−4.45 to −1.75)	−1.59 (−3.21 to 0.03)	−1.93 (−3.55 to −0.31)	−0.31 (−2.12 to 1.51)	0.22 (−1.72 to 2.16)	−0.07 (−1.59 to 1.45)	—

Each cell is the row treatment minus the column treatment in kilograms with 95% CI; negative values favour the row treatment.

### 3.8 Other outcomes

Fat-free mass was maintained or increased in most resistance-training trials. Tinsley 2019 reported increases of approximately 2–3% across groups, while Moro, 2016; Stratton, 2020; Moro 2020; and both Correia crossover trials found no material loss attributable to TRE (8, 17–21). Cui, 2025 reported a decrease with TRE alone, an increase with resistance training alone, and maintenance with the combined intervention (22).

Body-fat percentage and body weight generally declined when TRE created an additional energy deficit. Moro 2020 reported a 1.19-percentage-point greater reduction in body-fat percentage during cycling training (19). The Correia running crossover trial found no additional body-composition or aerobic-capacity benefit from TRE (21).

Metabolic outcomes were reported less consistently. Haganes, 2022 found improved glycated hemoglobin in the combined TRE and HIIT arm (10). Ameur, 2024 reported larger changes in fasting glucose, insulin and HOMA-IR with TRE plus high-intensity functional training than with either component alone (23). Dai, 2025 reported improvements in fat mass and related metabolic measures with flexible TRE plus aerobic exercise (24).

Performance outcomes were modality-specific. Resistance-training trials generally preserved or improved strength. Endurance trials reported either similar improvements under TRE and control diets or improved power relative to body weight after weight reduction (19, 21, 25).

### 3.9 Subgroup, sensitivity, and small-study analyses

Prespecified analyses were evaluated by exercise modality, eating-window duration, intervention duration, BMI status, sex, training status, metabolic risk, and exercise supervision. The available connected data supported modality-specific node estimates but not stable within-node meta-regression. Excluding crossover trials and linked follow-up reports did not change the primary model because those reports did not contribute duplicate observations. The pairwise estimate remained stable across change-score correlation assumptions.

Fewer than 10 independent studies contributed to the primary comparison. A comparison-adjusted funnel plot and regression test were therefore not used, in accordance with the prespecified threshold.

### 3.10 Certainty of evidence

See Table 7 here.

**Table 7:** GRADE/CINeMA certainty assessment.

Outcome/comparison	Within-study bias	Indirectness	Imprecision	Incoherence	Certainty	Rationale
TRE plus exercise vs. the same exercise: fat mass	Serious	Not serious	Serious	Not serious	Low	Several variances were reconstructed; most trials had some concerns.
TRE + resistance vs. exercise	Serious	Some indirectness	Serious	Not serious	Low	Four trials, but populations and energy control differed.
TRE + concurrent vs. exercise	Serious	Serious	Serious	Not serious	Low	Two trials with different functional programmes.
TRE + aerobic vs. exercise	Not serious	Serious	Serious	Not estimable	Low	One four-arm trial.
TRE + HIIT vs. exercise	Not serious	Serious	Serious	Not estimable	Low	One four-arm trial.
Relative ranking among combined modalities	Serious	Very serious	Serious	Not estimable	Very low	No direct head-to-head comparison between combined modalities.
Fat-free mass	Serious	Serious	Serious	Not estimable	Low	Direction was consistent but measures and variances were heterogeneous.
Glycemic and performance outcomes	Serious	Serious	Very serious	Not estimable	Very low	Few trials used common outcome definitions.

## 4 Discussion

### 4.1 Principal findings

The updated pairwise analysis found that TRE added an average fat-mass reduction of approximately 1.8 kg when participants completed the same exercise programme. The estimate was consistent across correlation assumptions and was similar in magnitude to prior pairwise reviews (3–5).

The network ranked concurrent training, HIIT and aerobic exercise above resistance training for fat-mass reduction. These rankings were probabilistic. Their confidence intervals overlapped, and the rankogram showed substantial uncertainty in the ordering of the active combined modalities.

Resistance training had the largest repeated evidence base among the combined nodes and consistently preserved strength and fat-free mass. This distinction matters because exercise selection should reflect the intended outcome rather than fat-mass ranking alone.

### 4.2 Practical implications

For adults who were overweight or obese and who prioritised fat-mass reduction and glycaemic improvement, TRE combined with aerobic exercise, HIIT, or a mixed programme may be considered. Exercise tolerance, supervision, musculoskeletal health, and medical risk should guide the choice.

For trained adults and people who prioritise lean-mass and strength preservation, TRE combined with resistance training is supported by the most repeated direct evidence. Adequate protein and total energy intake remain important.

### 4.3 Interpretation of certainty

The direct pooled fat-mass effect was rated low certainty because several variances required reconstruction and most trials had some RoB 2 concerns. Comparative modality rankings were rated very low certainty because no trial directly randomised participants between two TRE-plus-exercise modalities. The conclusions therefore describe higher probabilities of benefit rather than an optimal intervention.

## 5 Conclusion

TRE combined with exercise reduced fat mass more than the same exercise without TRE. Concurrent training, HIIT, and aerobic exercise had the highest probabilities of benefit for fat mass, while resistance training had the strongest repeated evidence for preserving strength and fat-free mass. The active combined modalities did not differ precisely from one another. Exercise modality should therefore be selected according to the person’s primary outcome, training experience, and capacity to adhere.

## Data Availability

The original contributions presented in the study are included in the article/Supplementary material, further inquiries can be directed to the corresponding author/s.
