# Supplementary material for: Comparative efficacy of time-restricted eating combined with different exercise modalities for improving body composition, metabolic health, and physical performance in adults: a systematic review and fat-mass network meta-analysis
Source: Front Nutr. 2026 Oct 27;13:1966901. doi: 10.3389/fnut.2026.1966901 (PMC13747753; doi:10.3389/fnut.2026.1966901)
Supplement: Supplementary file 1 [file Supplementary_file_1.docx]

Supplementary Materials

# Supplementary Table S1. PRISMA 2020 and PRISMA-NMA checklists

S1(a). PRISMA 2020 checklist

| **Item** | **Topic** | **Requirement addressed** | **Location** |
| --- | --- | --- | --- |
| 1 | Title | Identifies the report as a systematic review and network meta-analysis. | Title |
| 2 | Abstract | Structured summary with sources, eligibility, synthesis, effects, heterogeneity and certainty. | Abstract |
| 3 | Rationale | Context and rationale described. | Introduction |
| 4 | Objectives | Seven-node comparative question specified. | Introduction |
| 5 | Eligibility criteria | Population, interventions, comparators, outcomes and designs specified. | Methods 2.2 |
| 6 | Information sources | Five core databases and supplementary sources reported with final date. | Methods 2.3; Table S3 |
| 7 | Search strategy | Full platform-adapted strategies provided. | Table S3 |
| 8 | Selection process | Two-stage screening and conflict resolution described. | Methods 2.3 |
| 9 | Data collection | Extraction fields and trial linkage described. | Methods 2.4 |
| 10a | Data items—outcomes | Primary and secondary outcomes listed. | Methods 2.5 |
| 10b | Other variables | Effect modifiers and intervention dose variables listed. | Methods 2.4 and 2.7 |
| 11 | Risk of bias | RoB 2 methods and outcome-level forms provided. | Methods 2.6; Table S6 |
| 12 | Effect measures | MD and 95% CI specified. | Methods 2.7 |
| 13a–f | Synthesis methods | Eligibility for synthesis, data preparation, models, heterogeneity, sensitivity and reporting described. | Methods 2.5–2.7 |
| 14 | Reporting bias | Prespecified threshold for small-study assessment stated. | Methods 2.7; Results 3.9 |
| 15 | Certainty | GRADE/CINeMA methods specified. | Methods 2.8 |
| 16a–b | Study selection | PRISMA flow and exclusion reasons provided. | Figure 1; Table S2 |
| 17 | Study characteristics | Unique-trial and linked-report table provided. | Table 4; Table S4 |
| 18 | Risk of bias results | Domain and overall judgements reported. | Figure 2; Table S6 |
| 19–20 | Results of syntheses | Pairwise and network estimates, CIs, heterogeneity and sensitivity reported. | Results 3.4–3.9 |
| 21 | Reporting biases | Reason formal assessment was not activated reported. | Results 3.9 |
| 22 | Certainty | Certainty table provided. | Table 7 |
| 23a–d | Discussion | Interpretation, evidence context and implications reported. | Discussion |
| 24a–c | Registration and protocol | PROSPERO number and protocol status reported. | Methods 2.1 |
| 25 | Support | No funding was received. | Declarations |
| 26 | Competing interests | No competing interests. | Declarations |
| 27 | Availability | Analytic workbook supplied with the revision package. | Data availability statement |

**S1(b). PRISMA extension for network meta-analysis**

| **Section / topic** | **Item** | **Checklist requirement** | **Manuscript location** |
| --- | --- | --- | --- |
| **TITLE** | | | |
| Title | 1 | Identify the report as a systematic review incorporating a network meta-analysis (or related form of meta-analysis). | Title |
| **ABSTRACT** | | | |
| Structured summary | 2 | Provide a structured summary including, as applicable:  Background: main objectives Methods: data sources; study eligibility criteria, participants, and interventions; study appraisal; and synthesis methods, such as network meta-analysis.  Results: number of studies and participants identified; summary estimates with corresponding confidence/credible intervals; treatment rankings may also be discussed. Authors may choose to summarize pairwise comparisons against a chosen treatment included in their analyses for brevity. Discussion/Conclusions: limitations; conclusions and implications of findings. Other: primary source of funding; systematic review registration number with registry name. | Abstract |
| **INTRODUCTION** | | | |
| Rationale | 3 | Describe the rationale for the review in the context of what is already known, including mention of why a network meta-analysis has been conducted. | Introduction |
| Objectives | 4 | Provide an explicit statement of questions being addressed, with reference to participants, interventions, comparisons, outcomes, and study design (PICOS). | Introduction; Methods 2.2 |
| **METHODS** | | | |
| Protocol and registration | 5 | Indicate whether a review protocol exists and if and where it can be accessed (e.g., Web address); and, if available, provide registration information, including registration number. | Methods 2.1; Protocol registration |
| Eligibility criteria | 6 | Specify study characteristics (e.g., PICOS, length of follow-up) and report characteristics (e.g., years considered, language, publication status) used as criteria for eligibility, giving rationale. Clearly describe eligible treatments included in the treatment network, and note whether any have been clustered or merged into the same node (with justification). | Methods 2.2; 2.5 |
| Information sources | 7 | Describe all information sources (e.g., databases with dates of coverage, contact with study authors to identify additional studies) in the search and date last searched. | Methods 2.3; Table 2 |
| Search | 8 | Present full electronic search strategy for at least one database, including any limits used, such that it could be repeated. | Methods 2.3; Supplementary Table S3 |
| Study selection | 9 | State the process for selecting studies (i.e., screening, eligibility, included in systematic review, and, if applicable, included in the meta-analysis). | Methods 2.3; Results 3.1; Figure 1 |
| Data collection process | 10 | Describe method of data extraction from reports (e.g., piloted forms, independently, in duplicate) and any processes for obtaining and confirming data from investigators. | Methods 2.3–2.4 |
| Data items | 11 | List and define all variables for which data were sought (e.g., PICOS, funding sources) and any assumptions and simplifications made. | Methods 2.4–2.5; Supplementary Table S11 |
| Geometry of the network | S1 | Describe methods used to explore the geometry of the treatment network under study and potential biases related to it. This should include how the evidence base has been graphically summarized for presentation, and what characteristics were compiled and used to describe the evidence base to readers. | Methods 2.5; Results 3.5; Figure 4 |
| Risk of bias within individual studies | 12 | Describe methods used for assessing risk of bias of individual studies (including specification of whether this was done at the study or outcome level), and how this information is to be used in any data synthesis. | Methods 2.6; Results 3.3; Supplementary Table S6 |
| Summary measures | 13 | State the principal summary measures (e.g., risk ratio, difference in means). Also describe the use of additional summary measures assessed, such as treatment rankings and surface under the cumulative ranking curve (SUCRA) values, as well as modified approaches used to present summary findings from meta-analyses. | Methods 2.7; Results 3.4–3.7 |
| Planned methods of analysis | 14 | Describe the methods of handling data and combining results of studies for each network meta-analysis. This should include, but not be limited to:  Handling of multi-arm trials; Selection of variance structure; Selection of prior distributions in Bayesian analyses; and  Assessment of model fit. | Methods 2.7; Supplementary Tables S5, S7–S10 |
| Assessment of Inconsistency | S2 | Describe the statistical methods used to evaluate the agreement of direct and indirect evidence in the treatment network(s) studied. Describe efforts taken to address its presence when found. | Methods 2.7; Results 3.5, 3.6 |
| Risk of bias across studies | 15 | Specify any assessment of risk of bias that may affect the cumulative evidence (e.g., publication bias, selective reporting within studies). | Methods 2.6–2.8; Results 3.9 |
| Additional analyses | 16 | Describe methods of additional analyses if done, indicating which were pre-specified. This may include, but not be limited to, the following:  Sensitivity or subgroup analyses; Meta-regression analyses;  Alternative formulations of the treatment network; and Use of alternative prior distributions for Bayesian analyses (if applicable). | Methods 2.7; Results 3.9; Supplementary Table S7 |
| **RESULTS†** | | | |
| Study selection | 17 | Give numbers of studies screened, assessed for eligibility, and included in the review, with reasons for exclusions at each stage, ideally with a flow diagram. | Results 3.1; Figure 1; Supplementary Table S2 |
| Presentation of network structure | S3 | Provide a network graph of the included studies to enable visualization of the geometry of the treatment network. | Results 3.5; Figure 4 |
| Summary of network geometry | S4 | Provide a brief overview of characteristics of the treatment network. This may include commentary on the abundance of trials and randomized patients for the different interventions and pairwise comparisons in the network, gaps of evidence in the treatment network, and potential biases reflected by the network structure. | Results 3.5; Figure 4 |
| Study characteristics | 18 | For each study, present characteristics for which data were extracted (e.g., study size, PICOS, follow-up period) and provide the citations. | Results 3.2; Table 4; Supplementary Table S4 |
| Risk of bias within studies | 19 | Present data on risk of bias of each study and, if available, any outcome level assessment. | Results 3.3; Figure 2; Supplementary Table S6 |
| Results of individual studies | 20 | For all outcomes considered (benefits or harms), present, for each study: 1) simple summary data for each intervention group, and 2) effect estimates and confidence intervals. Modified approaches may be needed to deal with information from larger networks. | Results 3.2, 3.4, 3.8; Table 4; Supplementary Table S11 |
| Synthesis of results | 21 | Present results of each meta-analysis done, including confidence/credible intervals. In larger networks, authors may focus on comparisons versus a particular comparator (e.g. placebo or standard care), with full findings presented in an appendix. League tables and forest plots may be considered to summarize pairwise comparisons. If additional summary measures were explored (such as treatment rankings), these should also be presented. | Results 3.4–3.7; Figures 3–8; Tables 5–6; Supplementary Tables S5, S8–S10 |
| Exploration for inconsistency | S5 | Describe results from investigations of inconsistency. This may include such information as measures of model fit to compare consistency and inconsistency models, P values from statistical tests, or summary of inconsistency estimates from different parts of the treatment network. | Results 3.5–3.6 |
| Risk of bias across studies | 22 | Present results of any assessment of risk of bias across studies for the evidence base being studied. | Results 3.9 |
| Results of additional analyses | 23 | Give results of additional analyses, if done (e.g., sensitivity or subgroup analyses, meta-regression analyses, alternative network geometries studied, alternative choice of prior distributions for Bayesian analyses, and so forth). | Results 3.9; Supplementary Table S7 |
| **DISCUSSION** | | | |
| Summary of evidence | 24 | Summarize the main findings, including the strength of evidence for each main outcome; consider their relevance to key groups (e.g., healthcare providers, users, and policy-makers). | Discussion 4.1–4.3; Table 7 |
| Limitations | 25 | Discuss limitations at study and outcome level (e.g., risk of bias), and at review level (e.g., incomplete retrieval of identified research, reporting bias). Comment on the validity of the assumptions, such as transitivity and consistency. Comment on any concerns regarding network geometry (e.g., avoidance of certain comparisons). | Results 3.5; Discussion 4.3 |
| Conclusions | 26 | Provide a general interpretation of the results in the context of other evidence, and implications for future research. | Discussion 4; Conclusion 5 |
| **FUNDING** | | | |
| Funding | 27 | Describe sources of funding for the systematic review and other support (e.g., supply of data); role of funders for the systematic review. This should also include information regarding whether funding has been received from manufacturers of treatments in the network and/or whether some of the authors are content experts with professional conflicts of interest that could affect use of treatments in the network. | Declarations: Funding |

# Supplementary Table S2. Full-text exclusions

| **Report/category** | **Primary reason for exclusion** |
| --- | --- |
| Alternate-day fasting combined with aerobic exercise | Fasting regimen was alternate-day fasting rather than TRE. |
| Ramadan fasting and resistance/concurrent training reports | Religious fasting pattern did not meet the prespecified TRE definition. |
| Intermittent energy restriction plus sprint interval training | Diet was intermittent energy restriction rather than a daily TRE window. |
| Ribeiro 2021 | Participants were physically active, but no prospectively assigned exercise intervention or standardised exercise comparator was provided. |
| Isenmann 2021 | Exercise was a background habitual behaviour rather than a randomised or standardised exercise intervention. |
| Correia 2021 supramaximal performance report | The protocol assessed performance under feeding conditions without a longitudinal exercise-training intervention meeting the minimum duration criterion. |
| Haganes 2025 nutrient-intake secondary analysis | The report did not contribute a prespecified body-composition, metabolic-health or physical-performance outcome. |
| Hofstätter 2025 psychological-outcomes report | The report contained psychological outcomes only and did not contribute a prespecified clinical or performance outcome. |
| Zhou 2026 low-carbohydrate TRE plus sprint training | The low-carbohydrate co-intervention prevented assignment to a prespecified TRE node. |
| Sujan 2025 preconception/pregnancy trial | The pregnancy-specific population and gestational outcomes fell outside the adult general-health scope. |
| Jóźwiak 2025 | The allocation was quasi-randomised and did not meet the randomised-trial criterion. |
| Acute fasted-versus-fed exercise experiments | The exposure was acute or shorter than two weeks. |
| Ramadan exercise-timing studies | The intervention altered exercise timing during Ramadan rather than testing TRE plus an exercise modality. |
| Conference abstracts without complete data | A full report with extractable outcome data was unavailable. |

# Supplementary Table S3. Complete search strategies

## PubMed/MEDLINE

("time-restricted eating"[tiab] OR "time restricted eating"[tiab] OR "time-restricted feeding"[tiab] OR "time restricted feeding"[tiab] OR TRE[tiab] OR TRF[tiab] OR "intermittent fasting"[tiab] OR "intermittent energy restriction"[MeSH Terms] OR Fasting[MeSH Terms] OR "restricted feeding window"[tiab] OR "eating window"[tiab] OR "feeding window"[tiab] OR "16:8"[tiab] OR "14:10"[tiab] OR "18:6"[tiab]) AND ("exercise"[MeSH Terms] OR exercise[tiab] OR "resistance training"[MeSH Terms] OR "resistance training"[tiab] OR "strength training"[tiab] OR "weight training"[tiab] OR "aerobic exercise"[tiab] OR "endurance training"[tiab] OR "high-intensity interval training"[MeSH Terms] OR HIIT[tiab] OR "concurrent training"[tiab] OR "physical activity"[MeSH Terms] OR "physical activity"[tiab] OR "training program*"[tiab]) AND ("randomized controlled trial"[Publication Type] OR "randomized controlled trial"[tiab] OR "randomised controlled trial"[tiab] OR RCT[tiab] OR randomized[tiab] OR randomised[tiab] OR crossover[tiab] OR "cross-over"[tiab] OR "controlled clinical trial"[Publication Type])

## Web of Science

TS=("time-restricted eating" OR "time restricted eating" OR "time-restricted feeding" OR "time restricted feeding" OR TRE OR TRF OR "intermittent fasting" OR "eating window" OR "feeding window" OR "16:8" OR "14:10" OR "18:6") AND TS=(exercise OR "resistance training" OR "strength training" OR "weight training" OR "aerobic exercise" OR "endurance training" OR "high-intensity interval training" OR HIIT OR "concurrent training" OR "physical activity" OR "training program*") AND TS=(randomi* OR RCT OR crossover OR "cross-over" OR "controlled trial")

## Scopus

TITLE-ABS-KEY("time-restricted eating" OR "time restricted eating" OR "time-restricted feeding" OR "time restricted feeding" OR TRE OR TRF OR "intermittent fasting" OR "eating window" OR "feeding window" OR "16:8" OR "14:10" OR "18:6") AND TITLE-ABS-KEY(exercise OR "resistance training" OR "strength training" OR "weight training" OR "aerobic exercise" OR "endurance training" OR "high-intensity interval training" OR HIIT OR "concurrent training" OR "physical activity" OR "training program*") AND TITLE-ABS-KEY(randomi* OR RCT OR crossover OR "cross-over" OR "controlled trial") AND (LIMIT-TO(LANGUAGE,"English") AND LIMIT-TO(DOCTYPE,"ar"))

## Embase

('time restricted eating':ti,ab,kw OR 'time restricted feeding':ti,ab,kw OR tre:ti,ab,kw OR trf:ti,ab,kw OR 'intermittent fasting':ti,ab,kw OR 'eating window':ti,ab,kw OR 'feeding window':ti,ab,kw OR '16:8':ti,ab,kw OR '14:10':ti,ab,kw OR '18:6':ti,ab,kw OR 'fasting'/exp) AND ('exercise'/exp OR exercise:ti,ab,kw OR 'resistance training'/exp OR 'resistance training':ti,ab,kw OR 'strength training':ti,ab,kw OR 'aerobic exercise':ti,ab,kw OR 'endurance training':ti,ab,kw OR 'high intensity interval training'/exp OR hiit:ti,ab,kw OR 'concurrent training':ti,ab,kw OR 'physical activity'/exp) AND ('randomized controlled trial'/exp OR random*:ti,ab,kw OR rct:ti,ab,kw OR crossover:ti,ab,kw OR 'cross over':ti,ab,kw) AND [english]/lim AND [article]/lim

## Cochrane Library

([mh Fasting] OR "time-restricted eating":ti,ab,kw OR "time restricted eating":ti,ab,kw OR "time-restricted feeding":ti,ab,kw OR TRE:ti,ab,kw OR TRF:ti,ab,kw OR "intermittent fasting":ti,ab,kw OR "eating window":ti,ab,kw OR "feeding window":ti,ab,kw) AND ([mh Exercise] OR [mh "Resistance Training"] OR exercise:ti,ab,kw OR "resistance training":ti,ab,kw OR "strength training":ti,ab,kw OR "aerobic exercise":ti,ab,kw OR "endurance training":ti,ab,kw OR HIIT:ti,ab,kw OR "concurrent training":ti,ab,kw OR "physical activity":ti,ab,kw) AND ([mh "Randomized Controlled Trials as Topic"] OR random*:ti,ab,kw OR RCT:ti,ab,kw OR crossover:ti,ab,kw OR "cross-over":ti,ab,kw)

## Google Scholar

allintitle: ("body composition" OR metabolic OR performance) ("time restricted eating" OR "time restricted feeding") exercise randomized

Search counts: PubMed/MEDLINE 372; Web of Science 465; Scopus 506; Embase 603; Cochrane Library 207; Google Scholar 3,596. Total records identified: 5,749. Searches covered 2016 to 20 July 2026.

# Supplementary Table S4. Unique trial and report linkage

| **Unique ID** | **Trial** | **Report(s)** | **Design** | **Sample** | **Population** | **TRE** | **Exercise** | **Duration** | **Analytic treatment** |
| --- | --- | --- | --- | --- | --- | --- | --- | --- | --- |
| T1 | Moro cohort (8,9) | Moro 2016 primary; Moro 2021 long-term follow-up | Parallel RCT | 34 randomised; 20 at 12-month follow-up | Resistance-trained men | 8 h daily | Resistance training, 3 sessions/week | 8 weeks; linked 12-month follow-up | Primary 8-week report used in quantitative model; follow-up linked, not double-counted |
| T2 | Tinsley 2017 (26) | Primary report | Parallel RCT | 28 randomised; 18 analysed | Recreationally active men | 4 h on 4 days/week | Resistance training, 3 sessions/week | 8 weeks | Fat-mass change and imputed variance used |
| T3 | Tinsley 2019 (17) | Primary report | Three-arm RCT | 40 ITT; 24 per protocol | Resistance-trained women | ≈7.5 h daily | Supervised resistance training | 8 weeks | ITT retained; no compatible arm-level variance for primary network |
| T4 | Stratton 2020 (18) | Primary report | Parallel RCT | 26 | Recreationally active men | 8 h daily | Supervised full-body resistance training | 4 weeks | Fat-mass change used; change variance imputed |
| T5 | Kotarsky 2021 (27) | Primary report | Parallel RCT | 21 completers | Adults with overweight/obesity | 12:00–20:00 | Aerobic plus supervised resistance training | 8 weeks | Fat-mass contrast used |
| T6 | Moro 2020 (19) | Primary report | Parallel RCT | 16 | Elite male cyclists | 10:00–18:00 | Endurance cycling | 4 weeks | Body-fat percentage and performance synthesis |
| T7 | Brady 2021 (25) | Primary report | Parallel RCT | 17 | Male middle-/long-distance runners | 12:00–20:00 | Endurance running | 8 weeks | Body composition and running outcomes |
| T8 | Martínez-Rodríguez 2021 (28) | Primary report | Randomised trial | Active women | Active women | TRE/IF protocol | HIIT | 8 weeks | Body composition and performance synthesis |
| T9 | TREHIIT (10,11) | Haganes 2022 primary; Haganes 2025 follow-up | Four-arm RCT | 131 primary | Women with overweight/obesity | ≤10 h daily | HIIT, 3 sessions/week | 7 weeks; linked 2-year follow-up | Primary report used in network; follow-up linked |
| T10 | Correia 2023 (20) | Primary report | Randomised crossover | Adult men | Healthy trained men | 8 h daily | Resistance training | 4 weeks/condition | Paired synthesis; not entered without paired variance |
| T11 | Correia 2024 (21) | Primary report | Randomised crossover | 15 analysed | Male recreational runners | 13:00–21:00 | Structured endurance running | 4 weeks/condition | Paired synthesis; not entered without paired variance |
| T12 | Liu 2023 (29) | Primary report | Four-arm RCT | 77 | Female university students with hidden obesity | TRE protocol | Walking exercise | 8 weeks | Body composition synthesis; exact compatible change variance unavailable |
| T13 | Ameur 2024 (23) | Primary report | Three-arm RCT | 64 | Inactive women with obesity | ≤8 h daily | HIFT, 3 sessions/week | 12 weeks | Fat-mass contrast used with conservative imputed variance |
| T14 | Cui 2025 (22) | Primary report | Four-arm RCT | Young adults with overweight/obesity | Young adults with overweight/obesity | 10 h daily | Resistance training | 8 weeks | Reported fat-mass changes used |
| T15 | Dai 2025 (24) | Primary report | Four-arm assessor-blinded RCT | 104 ITT | Women aged 40–60 with overweight/obesity | Flexible 8 h daily | Aerobic exercise | 12 weeks | Adjusted 99% CI contrasts used |

# Supplementary Table S5. Pairwise analytic inputs

| **Study** | **Comparison** | **MD kg** | **SE** | **95% CI** | **Weight** | **Data status** |
| --- | --- | --- | --- | --- | --- | --- |
| Moro 2016 | TRE+resistance vs exercise | -1.31 | 0.561 | -2.41 to -0.21 | 19.9% | reported/reconstructed change contrast |
| Tinsley 2017 | TRE+resistance vs exercise | -1.40 | 2.959 | -7.20 to 4.40 | 0.7% | change variance imputed |
| Stratton 2020 | TRE+resistance vs exercise | -0.10 | 3.306 | -6.58 to 6.38 | 0.6% | change SD imputed with r=0.50 |
| Kotarsky 2021 | TRE+concurrent vs exercise | -2.00 | 0.556 | -3.09 to -0.91 | 20.3% | SE reconstructed from interaction information |
| Haganes 2022 | TRE+HIIT vs HIIT | -1.60 | 0.689 | -2.95 to -0.25 | 13.2% | adjusted contrast reconstructed from reported arm effects |
| Ameur 2024 | TRE+concurrent vs exercise | -5.40 | 2.560 | -10.42 to -0.38 | 1.0% | change SD imputed with r=0.50; conservative SE |
| Cui 2025 | TRE+resistance vs exercise | -2.10 | 0.640 | -3.36 to -0.84 | 15.3% | reported changes; values treated as SEs |
| Dai 2025 | TRE+aerobic vs exercise | -2.01 | 0.466 | -2.92 to -1.10 | 28.9% | 99% CI converted to SE |

# Supplementary Table S6. RoB 2 outcome-level judgements and rationales

| **Trial** | **D1** | **D2** | **D3** | **D4** | **D5** | **Overall** | **Rationale** |
| --- | --- | --- | --- | --- | --- | --- | --- |
| Moro 2016 | SC | SC | L | L | SC | SC | Objective body-composition measurement; one or more domains lacked complete information on allocation concealment, attrition handling or prespecified outcome analysis. |
| Tinsley 2017 | SC | SC | SC | L | SC | SC | Objective body-composition measurement; one or more domains lacked complete information on allocation concealment, attrition handling or prespecified outcome analysis. |
| Tinsley 2019 | L | L | L | L | L | L | Objective body-composition measurement; randomisation, missing data and reporting were sufficiently described. |
| Stratton 2020 | L | L | L | L | L | L | Objective body-composition measurement; randomisation, missing data and reporting were sufficiently described. |
| Kotarsky 2021 | SC | SC | SC | L | L | SC | Objective body-composition measurement; one or more domains lacked complete information on allocation concealment, attrition handling or prespecified outcome analysis. |
| Moro 2020 | SC | SC | L | L | SC | SC | Objective body-composition measurement; one or more domains lacked complete information on allocation concealment, attrition handling or prespecified outcome analysis. |
| Brady 2021 | SC | L | L | L | SC | SC | Objective body-composition measurement; one or more domains lacked complete information on allocation concealment, attrition handling or prespecified outcome analysis. |
| Martínez-Rodríguez 2021 | SC | SC | SC | L | SC | SC | Objective body-composition measurement; one or more domains lacked complete information on allocation concealment, attrition handling or prespecified outcome analysis. |
| Haganes 2022 | L | L | L | L | L | L | Objective body-composition measurement; randomisation, missing data and reporting were sufficiently described. |
| Correia 2023 | SC | SC | L | L | SC | SC | Objective body-composition measurement; one or more domains lacked complete information on allocation concealment, attrition handling or prespecified outcome analysis. |
| Correia 2024 | SC | L | L | L | SC | SC | Objective body-composition measurement; one or more domains lacked complete information on allocation concealment, attrition handling or prespecified outcome analysis. |
| Liu 2023 | SC | SC | L | L | SC | SC | Objective body-composition measurement; one or more domains lacked complete information on allocation concealment, attrition handling or prespecified outcome analysis. |
| Ameur 2024 | L | SC | L | L | SC | SC | Objective body-composition measurement; one or more domains lacked complete information on allocation concealment, attrition handling or prespecified outcome analysis. |
| Cui 2025 | SC | L | L | L | SC | SC | Objective body-composition measurement; one or more domains lacked complete information on allocation concealment, attrition handling or prespecified outcome analysis. |
| Dai 2025 | L | L | L | L | L | L | Objective body-composition measurement; randomisation, missing data and reporting were sufficiently described. |

A second-reviewer verification sheet is included below.

| **Trial** | **Reviewer 1 initials/date** | **Reviewer 2 initials/date** | **Consensus initials/date** |
| --- | --- | --- | --- |
| Moro 2016 | WK,16^th^ July | AB, 18^th^ July | WK/AB, 20^th^ July |
| Tinsley 2017 | WK,16^th^ July | AB, 18^th^ July | WK/AB, 20^th^ July |
| Tinsley 2019 | WK,16^th^ July | AB, 18^th^ July | WK/AB, 20^th^ July |
| Stratton 2020 | WK,16^th^ July | AB, 18^th^ July | WK/AB, 20^th^ July |
| Kotarsky 2021 | WK,16^th^ July | AB, 18^th^ July | WK/AB, 20^th^ July |
| Moro 2020 | WK,16^th^ July | AB, 18^th^ July | WK/AB, 20^th^ July |
| Brady 2021 | WK,16^th^ July | AB, 18^th^ July | WK/AB, 20^th^ July |
| Martínez-Rodríguez 2021 | WK,16^th^ July | AB, 18^th^ July | WK/AB, 20^th^ July |
| Haganes 2022 | WK,16^th^ July | AB, 18^th^ July | WK/AB, 20^th^ July |
| Correia 2023 | WK,16^th^ July | AB, 18^th^ July | WK/AB, 20^th^ July |
| Correia 2024 | WK,16^th^ July | AB, 18^th^ July | WK/AB, 20^th^ July |
| Liu 2023 | WK,16^th^ July | AB, 18^th^ July | WK/AB, 20^th^ July |
| Ameur 2024 | WK,16^th^ July | AB, 18^th^ July | WK/AB, 20^th^ July |
| Cui 2025 | WK,16^th^ July | AB, 18^th^ July | WK/AB, 20^th^ July |
| Dai 2025 | WK,16^th^ July | AB, 18^th^ July | WK/AB, 20^th^ July |

# Supplementary Table S7. Sensitivity analyses

| **Assumed correlation** | **Pooled MD** | **Lower95** | **Upper95** | **I2** | **Tau2** |
| --- | --- | --- | --- | --- | --- |
| 0.300 | -1.839 | -2.331 | -1.346 | 0.000 | 0.000 |
| 0.500 | -1.845 | -2.336 | -1.354 | 0.000 | 0.000 |
| 0.700 | -1.860 | -2.348 | -1.371 | 0.000 | 0.000 |

# Supplementary Table S8. Network direct contrasts

| **Study** | **Treatment A** | **Treatment B** | **A−B effect kg** | **SE** | **Data status** |
| --- | --- | --- | --- | --- | --- |
| Moro 2016 | TRE+RT | Exercise | -1.31 | 0.561 | direct |
| Tinsley 2017 | TRE+RT | Exercise | -1.40 | 2.959 | direct |
| Stratton 2020 | TRE+RT | Exercise | -0.10 | 3.306 | imputed change variance |
| Cui 2025 | TRE+RT | Exercise | -2.10 | 0.640 | reported changes |
| Kotarsky 2021 | TRE+Concurrent | Exercise | -2.00 | 0.556 | reconstructed SE |
| Ameur 2024 | TRE+Concurrent | Exercise | -5.40 | 2.560 | conservative imputed SE |
| Dai 2025 | TRE+Aerobic | Control | -2.85 | 0.450 | reported adjusted 99% CI |
| Dai 2025 | TRE+Aerobic | TRE | -1.56 | 0.458 | reported adjusted 99% CI |
| Dai 2025 | TRE+Aerobic | Exercise | -2.01 | 0.466 | reported adjusted 99% CI |
| Haganes 2022 | TRE | Control | -1.60 | 0.690 | reported/reconstructed adjusted effect |
| Haganes 2022 | Exercise | Control | -1.50 | 0.690 | reported/reconstructed adjusted effect |
| Haganes 2022 | TRE+HIIT | Control | -3.10 | 0.690 | reported/reconstructed adjusted effect |

# Supplementary Table S9. Rank probabilities

| **Treatment** | **Rank 1** | **Rank 2** | **Rank 3** | **Rank 4** | **Rank 5** | **Rank 6** | **Rank 7** |
| --- | --- | --- | --- | --- | --- | --- | --- |
| Control | 0.000 | 0.000 | 0.000 | 0.000 | 0.000 | 0.006 | 0.994 |
| TRE | 0.000 | 0.000 | 0.005 | 0.052 | 0.678 | 0.264 | 0.000 |
| Exercise | 0.000 | 0.000 | 0.000 | 0.006 | 0.265 | 0.724 | 0.005 |
| TRE+RT | 0.084 | 0.197 | 0.283 | 0.410 | 0.026 | 0.000 | 0.000 |
| TRE+Concurrent | 0.451 | 0.253 | 0.178 | 0.110 | 0.007 | 0.000 | 0.000 |
| TRE+Aerobic | 0.136 | 0.343 | 0.357 | 0.164 | 0.000 | 0.000 | 0.000 |
| TRE+HIIT | 0.329 | 0.207 | 0.176 | 0.258 | 0.024 | 0.006 | 0.000 |

# Supplementary Table S10. Contribution matrix

| **Node** | **Ameur 2024** | **Cui 2025** | **Dai 2025** | **Haganes 2022** | **Kotarsky 2021** | **Moro 2016** | **Stratton 2020** | **Tinsley 2017** |
| --- | --- | --- | --- | --- | --- | --- | --- | --- |
| TRE | 0.0 | 0.0 | 66.7 | 33.3 | 0.0 | 0.0 | 0.0 | 0.0 |
| Exercise | 0.0 | 0.0 | 66.4 | 33.6 | 0.0 | 0.0 | 0.0 | 0.0 |
| TRE+RT | 0.0 | 15.6 | 41.7 | 21.1 | 0.0 | 20.3 | 0.6 | 0.7 |
| TRE+Concurrent | 1.7 | 0.0 | 41.7 | 21.1 | 35.5 | 0.0 | 0.0 | 0.0 |
| TRE+Aerobic | 0.0 | 0.0 | 73.0 | 27.0 | 0.0 | 0.0 | 0.0 | 0.0 |
| TRE+HIIT | 0.0 | 0.0 | 0.0 | 100.0 | 0.0 | 0.0 | 0.0 | 0.0 |

# Registration and data availability

PROSPERO registration: CRD420261440609. The workbook supplied contains the pairwise inputs, network contrast matrix, league table, rank probabilities, contribution matrix, RoB 2 judgements, and PRISMA counts.

# Supplementary Table S11. Extractable quantitative data used in the fat-mass analyses

| **Study** | **Group-specific sample** | **Baseline/post or change data** | **Analytic contrast** | **Exercise dose, adherence and energy control** | **Exact analytic source** |
| --- | --- | --- | --- | --- | --- |
| Moro 2016 (8) | TRE+RT n=17; exercise n=17 | TRE: change −1.61 ± 1.53 kg; control: −0.30 ± 1.70 kg | MD −1.31 kg; SE 0.561 | Resistance training 3 sessions/week; energy and macronutrients matched | Primary report body-composition results |
| Tinsley 2017 (26) | TRE+RT and exercise groups; 18 analysed | TRE: 17.8 ± 7.8 to 17.2 ± 8.3 kg; control: 13.7 ± 4.5 to 14.5 ± 3.8 kg | MD −1.40 kg; change variance imputed | Resistance training 3 sessions/week; TRE applied 4 days/week | Primary report body-composition table |
| Stratton 2020 (18) | TRE+RT n=13; exercise n=13 | TRE: 16.8 ± 8.6 to 15.3 ± 8.5 kg; control: 16.3 ± 8.5 to 14.9 ± 8.1 kg | MD −0.10 kg; SE 3.306 using r=0.50 | Supervised full-body resistance training 3 sessions/week; 25% energy deficit; protein 1.8 g/kg/day | Primary report Section 3.3 |
| Kotarsky 2021 (27) | TRE+concurrent n=11; exercise n=10 | TRE: 33 ± 2 to 30 ± 2 kg; control: 33 ± 2 to 32 ± 2 kg; values are mean ± SE | MD −2.00 kg; SE 0.556 reconstructed | Resistance sessions on 3 non-consecutive days plus aerobic target; spontaneous energy reduction in both groups | Primary report Table 5 and interaction p=0.002 |
| Haganes 2022 (10) | TRE+HIIT n=32; HIIT n=33; TRE n=33; control n=33 | Adjusted effects versus control: TRE −1.6 kg; HIIT −1.5 kg; TRE+HIIT −3.1 kg | Combined vs HIIT MD −1.60 kg; SE 0.689 reconstructed | HIIT 3 sessions/week; ≤10 h eating window; high adherence | Primary report adjusted outcome model |
| Ameur 2024 (23) | TRE+HIFT n=20; HIFT n=24; TRE n=20 | Combined: 39.3 ± 9.2 to 31.0 ± 6.7 kg; HIFT: 35.8 ± 8.7 to 32.9 ± 8.6 kg | Change MD −5.40 kg; conservative SE 2.560 using r=0.50 | HIFT 3 sessions/week; ≤8 h eating window; calorie intake recorded | Primary report Table 2 |
| Cui 2025 (22) | Four-arm RCT; reported TRE+RT and RT changes | TRE+RT change −3.2 ± 0.4 kg; RT change −1.1 ± 0.5 kg | MD −2.10 kg; SE 0.640 treating reported uncertainty as SE | Resistance training with 10 h TRE | Primary report results and body-composition table |
| Dai 2025 (24) | 26 participants per arm; n=104 ITT | TRE+aerobic vs exercise MD −2.01 kg (99% CI −3.21 to −0.81); vs control −2.85 kg (−4.01 to −1.69) | SE 0.466 for combined vs exercise after conversion of 99% CI | 12-week aerobic exercise; flexible 8 h TRE; adherence 83%–87% | Primary report adjusted intention-to-treat model |
